# Emerging questions on the mechanisms and dynamics of 3D genome evolution in spiralians

**DOI:** 10.1093/bfgp/elad043

**Published:** 2023-10-09

**Authors:** Thea F Rogers, Oleg Simakov

**Affiliations:** Department of Neuroscience and Developmental Biology, Division of Molecular Evolution and Development, University of Vienna, Vienna, Austria; Department of Neuroscience and Developmental Biology, Division of Molecular Evolution and Development, University of Vienna, Vienna, Austria

**Keywords:** genome evolution, 3D genome organisation, Spiralia, gene regulation, chromatin interactions

## Abstract

Information on how 3D genome topology emerged in animal evolution, how stable it is during development, its role in the evolution of phenotypic novelties and how exactly it affects gene expression is highly debated. So far, data to address these questions are lacking with the exception of a few key model species. Several gene regulatory mechanisms have been proposed, including scenarios where genome topology has little to no impact on gene expression, and vice versa. The ancient and diverse clade of spiralians may provide a crucial testing ground for such mechanisms. Sprialians have followed distinct evolutionary trajectories, with some clades experiencing genome expansions and/or large-scale genome rearrangements, and others undergoing genome contraction, substantially impacting their size and organisation. These changes have been associated with many phenotypic innovations in this clade. In this review, we describe how emerging genome topology data, along with functional tools, allow for testing these scenarios and discuss their predicted outcomes.

## INTRODUCTION

**Figure 1:**
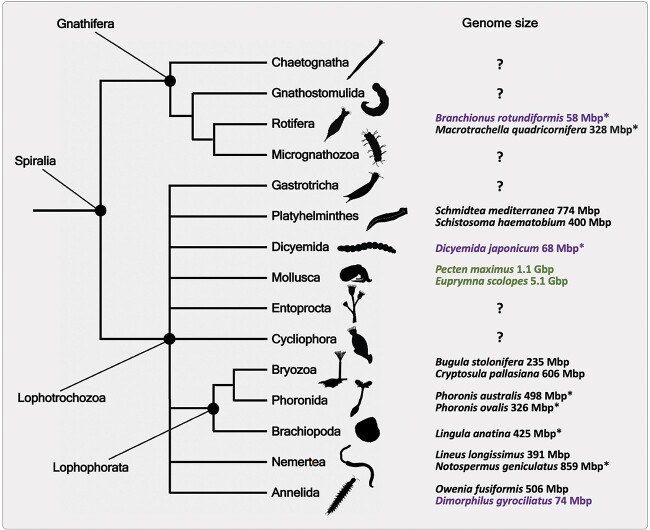
Spiralian phylogeny and genome sizes for selected species in each phylum. Genome sizes are listed for two species per clade unless there is only one genome assembly available for the phylum. A question mark signifies that the genome size is not known for any species in the clade. Purple indicates that the genome is under 100 Mbp and green indicates that the genome is over 1 Gbp. All assemblies are chromosome scale except for the ones with an asterisk which denotes a scaffold-level assembly. Images courtesy of PhyloPic.org except for Gnathostomulida, Micrognathozoa, Dicyemida, Entoprocta and Cycliophora, which were made using Adobe Illustrator. References for genome sizes are as follows: *B. rotundiformis* [23], *Macrotrachella quadricornifera* [111], *S. mediterranea* [11], *S. haematobium* [12], *D. japonicum* [24], *P. maximus* [8], *Euprymna scolopes* [27], *B. stolonifera* [13], *Cryptosula pallasiana* [14], *P. australis* [85], *P. ovalis* [112], *L. anatina* [113], *L. longissimus* [114], *N. geniculatus* [85], *N. geniculatus* [6], *O. fusiformis* [5], *Dinophilus gyrociliatus* [6].

Spiralia separated from the other major protostome lineage Ecdysozoa approximately 550 MYA [1], and is one of the most species-rich and morphologically diverse ancient animal clades [2]. This superphylum comprises several key species groups of mostly marine organisms such as molluscs, annelids, lophophorates, platyhelminthes and nemerteans (Figure 1). Difficulties in accessibility and laboratory rearing has resulted in few spiralians being investigated at the molecular level. Thus, general understanding regarding the genomic and regulatory mechanisms uniting this clade is lacking. Due to the emergence of several research groups over the past years, this gap has been consistently closing. The original, very limited studies that explore spiralian genomics [3, 4] have been extended to encompass representative species in other spiralian clades, including (but not limited to) polychaete worms [5, 6], molluscs [7–10], platyhelminthes, [11, 12], nemerteans [13, 14] and bryozoans [13, 14]. Furthermore, development and application of new molecular tools to spiralians such as HCR [15–18] and CRISPR-Cas9 [19–22] have finally enabled a peek into the variety of evolutionary mechanisms employed in this clade.

From an evolutionary perspective, one of the most fascinating aspects of spiralians is the diversity of their genomes. Ranging from the very small genomes of often parasitic or symbiotic species (e.g. less than 100 Mbp in some rotifer [23], dicyemid [24], annelid species [6]) to some of the largest invertebrate animal genomes (e.g. ~5 Gbp in cephalopod molluscs [10, 25]) (Figure 1). Due to many new chromosomal-scale genome assemblies emerging for spiralians, we can finally uncover broad genomic changes. Recent studies have revealed large-scale chromosomal element conservation in animal genomes, including many spiralians [26]. However, some spiralian genomes were shown to have undergone a substantial genome ‘restructuring’, e.g. cephalopods [10, 27]. Moreover, the genome expansion and contraction trends have been found to be largely driven by repetitive element expansions [25, 28, 29] with very few suggestions raised for the occurrence of whole genome duplication [30]. How different modes of spiralian genome evolution can result in novel gene regulation and eventually organismal novelties is the next emerging frontier in spiralian biology.

**Figure 2:**
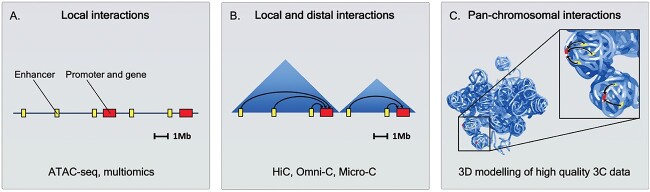
Methodology for studying 3D genome evolution and gene regulation. (**A**) ATAC-seq and multiomics (scATAC-seq and scRNA-seq) can be used to study chromatin interactions; however, a lack of information on TAD structures may mean that distal enhancers are missed in analyses of gene regulation. (**B**) Data on TAD structures (blue triangles) highlights whole contact regions, and therefore both local and distal interactions (black arrows) can be captured using 3C methods such as HiC, Omni-C and Micro-C. (**C**) If 3C data are of sufficient quality, these can also be used to model whole chromosomes in 3D. Consequently, the position of chromatin interactions and accessible sites can be viewed relative to the whole chromosome, revealing detailed information on structural organisation of genes and regulatory elements. The chromosome in (C) is 190 Mbp in size and based on the 3D chromosome model by Tereza Clarence in [27].

Across metazoans, *cis*-regulatory elements (CREs), including promoters, enhancers, silencers and operators, control the transcription of neighbouring genes. While some CREs are in close proximity to the genes they regulate, many are located further away and require precise folding of chromatin in order to perform their function. Chromatin interactions can be examined using assays for open chromatin such as ATAC-seq (assay for transposase-accessible chromatin) and multiomic (a combination of scATAC-seq and scRNA-seq) approaches (Figure 2A). Complementary to this, genome topology can be studied using Chromatin Conformation Capture (3C) techniques such as HiC, Omni-C and Micro-C, and accordingly contact maps of genome structure or 3D chromosome models can be generated (Figure 2B, C) [31]. 3C methods have revealed that in most animal genomes, chromosomes fold into 3D arrangements of chromatin, in which areas of high interaction frequency are termed topologically associating domains (TADs). TADs colocalise regions of the genome in order for CREs to control gene expression within them, and thus TAD boundaries are predicted to represent regions of the genome across which transcriptional regulation is impeded [32]. However, how changes in TAD structure and genome topology can result in alterations in gene expression is a debated topic [32, 33] that is heavily biased by investigations in few select model species. For instance, recent research in *Drosophila* [34, 35] and mammals [36] suggests that enhancers can act across TAD boundaries and some studies in *Drosophila* imply that distal enhancers are not always important in regulating gene expression within TADs [37]. Conversely, recent functional studies in mice show the importance of TAD structure in gene regulation during development [38]. The view from genomically and morphologically diverse groups such as those within the spiralian clade may thus provide a valuable new testing ground for these questions [32]. In this review, building on previous genomic and transcriptomic research and functional insights from various spiralian as well as non-spiralian models, we discuss the potential role of the emerging application of genome topology analyses in studies of spiralian evolution.

## SPIRALIANS AND THE ERA OF COMPLETE CHROMOSOMAL-SCALE GENOMES

The very first spiralian genomes [3, 4] were published over a decade ago. While revealing and already highlighting the genomic diversity of the clade, the scaffold and contig-level assemblies were insufficient to address large-scale evolutionary genome organisation questions. In recent years, the addition of early genome-wide 3C conformation capture methods has allowed the stitching together the previously assembled pieces to form chromosomal-level assemblies. While this method became the ‘golden’ standard, sometimes it was also complemented and validated by traditional genetic linkage information [39]. Together, this has been fruitful to reveal syntenic organisation of animal genomes [26, 40], including, most recently, spiralians [5]. Although this has also allowed for the incorporation of native HiC data to resolve TADs, the early protocols favoured coarse chromosomal contacts useful primarily for chromosomal assemblies and lacked sufficient resolution. As such, among spiralians, TADs have been studied only in a few cases, such as cephalopods [27].

Investigating genome topology at high resolution also requires high-accuracy, gap-free and, optimally, haplotype-resolved genomes. As the sequencing technologies improve, most of the earlier published spiralian genomes are due for an overhaul to fix many gaps in their assemblies. Recent developments in long-read sequencing methods, such as Oxford Nanopore and PacBio HiFi producing highly accurate reads, are necessary steps to generate a complete and gap-free assembly before HiC scaffolding. Such approaches only now become scale-able to large numbers of species, including the often neglected clades such as spiralians [41, 42]. The next big advancement in spiralian genome sequencing and assembly will be centred around the generation of such gap-free, highly accurate, and often haplotype-resolved, reference assemblies. While such assemblies are not expected to reveal novel topological features at the chromosomal or even TAD-level resolution, recent high-resolution conformational methods (e.g. Micro-C) may be applied to resolve local enhancer–promoter interactions that were otherwise masked in the previously unresolved gap-rich and often repetitive regions.

## GENOME COMPARTMENT FORMATION IN SPIRALIANS

To ascertain how genome topology drives the evolution of novel gene regulation, it is important to understand the mechanisms underlying 3D chromatin organisation. So far, two mechanisms have been proposed to drive TAD development. Firstly, chromatin can be divided into two distinct components based on its epigenetic properties. These are A compartments, which include areas of active chromatin, and B compartments, which consist of loci with inactive chromatin [43]. The second method of TAD formation, known as loop extrusion, includes the ring-shaped protein complex cohesin and the transcriptional repressor CTCF [44, 45]. In order to bring distant loci into close proximity and enable interaction, the cohesin ring attaches to the linear DNA sequence and reels in the chromatin fibre. This process is stopped when two CTCF-binding sites are encountered on either side of the DNA sequence and are convergently oriented (‘pointing’ inwards towards the constructed loop). TAD borders are subsequently established, and loci within these regions interact less frequently than those divided by inward-facing CTCF-binding sites [46].

The topological organisation of chromatin into A/B compartments is a conserved mechanism across eukaryotes. However, direct evidence indicates that loop extrusion is the primary method of TAD establishment vertebrates [46–49], reviewed in [32]. For example, the depletion of CTCF-binding sites [47–49] and components of the cohesin complex [46, 50] in various species causes the significant deterioration of most TAD structures, and, intriguingly, strengthening of A/B compartmentalisation. As of yet, direct evidence for TAD formation through either mechanism in invertebrates is lacking, and even indirect evidence in protostomes is limited [32]. The data that are available for this clade primarily focus on dipteran insects, including *Drosophila* [51] and *Anopheles* [52], both of which have genomes that feature CTCF-binding motifs. Nevertheless, chromatin organisation in these species is primarily regulated by A/B compartmentalisation. On the other hand, since cohesin protein complexes are conserved across eukaryotes [53] and CTCF originated in the bilaterian ancestor [54], one might expect loop extrusion to be the most common means of TAD configuration across bilaterians, and, accordingly, spiralians. Consistent with this, CTCF-binding sites were recently reported to be enriched at TAD borders in the cephalopod mollusc *Euprymna scolopes* [27]*.* This, to our knowledge, is the only study implicating the loop extrusion model in genome topology evolution in a spiralian. Taken together, the fragmented and conflicting evidence presented here is not enough to suggest a shared or divergent mode of compartment formation in the spiralian lineage, and further topology data across different spiralian groups are needed to decipher this.

## DISTRIBUTION OF GENES AND REGULATORY ELEMENTS IN A 3D SPACE

The main implication of topological organisation, as opposed to the ‘one-dimensional’ view of the genome, is that distal regulatory elements may play a central role in gene regulation, depending on their location in the 3D interaction space (Figure 2). As such, immediate genomic proximity may be less relevant and regulatory regions that are genomically more distant, but come physically close to promoters, become the major components of the gene regulatory network.

**Figure 3:**
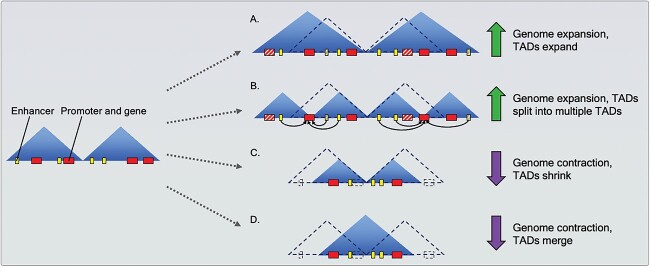
3D genome topology after genome expansion or contraction. The TADs (blue triangles) preceding the dotted arrows represent the ancestral genome state. Dashed triangles after the dotted arrows represent the ancestral TAD structure that has been lost during genome expansion (green arrows) or contraction (purple arrows). During genome expansion, TADs may (**A**) expand to form larger regulatory regions or (**B**) split into multiple TADs to form more regulatory ‘units’. Genome expansion is often accompanied by gene and/or regulatory element gain (striped rectangles). Solid black arrows in (B) represent interactions with promoters and genes that have moved to TAD boundaries during expansion and consequently span multiple TADs. As a result, enhancers in both neighbouring TADs have ‘access’ to them. Conversely, during genome contraction, TADs may (**C**) shrink to form smaller regulatory regions or (**D**) merge into one regulatory block. Genome contraction is often accompanied by gene and/or regulatory element loss (dashed rectangles).

Recent papers (e.g. [55]) have reported that genes and their regulatory regions show complex localisations within single TADs. While enhancers and target genes located at the opposite genomic ends of a given TAD may form tight interactions (held together by the loop extrusion mechanism), the sequences in-between (located centrally in the TAD) may evolve neutrally or be under different regulatory control. Moreover, the strength of this observation is likely to be vastly different, depending on the genome size and intergenic distances. While in some smaller genomes of model species, such as *Drosophila*, where the impact of topology on gene regulation has been studied in greater detail [56, 57], the effects are not as clear in larger genomes and the topological organisation may be a critical factor in gene regulation. In large genomes, particularly after a long evolutionary history of transposable element expansion, intergenic and intronic regions expand in correlated manner to the genome size [58], and absence of maintained genome topology would imply that any functional regulatory region must be located in the nearest vicinity of the gene. However, research thus far indicates that generally, TAD size correlates with genome size [59]; therefore, in the case of topological interactions, such distances may be extended substantially (Figure 3A, B). Among spiralians, the large genomes of cephalopods harbour correlatively large topological domains, slightly larger than the sizes observed in the human genome [27].

Genome expansion may have distinct effects on genome topology and gene regulation. For example, when TADs expand correlatively with the genome size, novel interactions will only happen due to new regulatory sequences emerging or translocating into the TAD (Figure 3A). The alternative scenario is the splitting of the original TAD into smaller units (Figure 3B). In this scenario, novel partitioning and interactions may arise. It may occur that the new TAD boundary emerges at or in close proximity to a gene, regulatory element or microsyntenic cluster. The gene, regulatory element or microsynteny may thus have interactions to separate topological domains. The end result may look similar to the scenario observed for the *HoxD* locus in the mouse [60], where the genes are differentially regulated during separate stages of limb development. While it is too early to indicate whether similar regulatory logic may be present in spiralian genomes, emerging evidence suggests the possibility of such ‘biphasic’ TADs in cephalopods in the evolution of some of their novel organs [61].

The opposite force of genome expansion is DNA loss and subsequent genome contraction. Several spiralian genomes have undergone genome contraction compared with either the ancestral state, for example, the mesozoan *Dicyema japonicum* [24] or the annelid model *Dimorphilus gyrociliatus* [6] (Figure 1) or show localised deletions that happen spontaneously in all genomes. In such cases, TADs can be predicted to shrink in size (Figure 3C) or undergo fusion (Figure 3D). While in the TAD shrinkage scenario the regulatory landscape will be substantially reduced but otherwise unaltered, in the fusion scenario the genes will be able to interact with regulatory regions that had been otherwise beyond their reach. Such scenario may again lead to *HoxD*-like ‘biphasic’ (or even ‘multiphasic’) regulatory interactions. Another possibility, given the very small genome size of these species, could also be the ‘disappearance’ of pervasive topology (due to CTCF loss), as is well known for the nematode *Caenorhabditis elegans* [62] (with a notable exception of the X chromosome [63]). In such small genomes, genes are comparatively closely positioned to each other so that the functional advantage of genome topology may be of no selective advantage and the regulation is accomplished through closest enhancer and promoter elements, which may involve operon-like gene cluster organisation [64]. While no genome topology data exist yet for these species, these predictions may soon become testable.

The highly diverse and dynamic genome sizes, especially in spiralians, may play a key role in influencing 3D chromatin organisation which, in turn, will define the regulatory interactions and the constraints of their expansion or contraction over the macro-evolutionary time-scale. While alterations to TADs due to translocations have been extensively discussed [32], this impact of genome size may prove to be another crucial, yet little investigated, factor. In some of the largest spiralian genomes, e.g. most cephalopod genomes reaching twice the size of the human genome, data on gene deserts and localisations to particular topological regions are only beginning to emerge [27]. As such, the insights from spiralian genomes may also help shed light into gene regulation in the many other enigmatic clades, including the largest animal genomes sequenced so far (axolotl [65] and lungfish [66]), as well as in animals that lack CTCF-driven persistent genome topology [54], where the regulatory interactions can be predicted to be very short [32, 67]. A signature of macro-evolutionarily persistent topology will then be non-random distribution of genes (in particular local, or microsyntenic, gene linkages) and gene deserts in relation to the genome topology and its evolutionary history. To this end, several such microsyntenic density signatures have been observed in animal genomes [68]. In addition, the presence of a higher number of maintained microsyntenic regions in bilaterians, as opposed to cnidarians [68], also correlates with the reported emergence of CTCF-driven topological organisation in bilaterians [54]. As such, amount and properties of retained microsyntenies can be a direct correlate of persistent genome topology maintained over larger evolutionary time-scales.

To further test these observations, several state-of-the-art techniques can be applied. In particular, the recent application of multiomic approaches can find correlated open chromatin regions with target genes. This opens up avenues to test whether the correlated putative regulatory regions are in the direct vicinity, closer to the opposite TAD border or uncorrelated with the potential target gene. In addition, emerging tools on 3D modelling (e.g. [69]) are crucial to reveal whether regulatory regions are situated on the chromosomal surface, suggesting potential interactions with the nuclear lamina or other chromosomes. This information is harder to access from 3C contact maps alone (Figure 2).

## TOWARDS UNDERSTANDING THE ROLE OF GENOME TOPOLOGY IN SPIRALIAN DEVELOPMENTAL PROCESSES

Spiralia includes many taxa that are highly diverse in their developmental or regenerative capabilities, such as planarian and annelid species that can regenerate every part of the body [70–72], as well as others such as rotifers and some mollusc species which have no or little ability to regenerate [70, 71]. Very few studies exist that link 3D genome evolution and spiralian development or regeneration; however, several potential modalities can be observed. One of the most well-studied developmental genes in spiralians are the Hox genes. Hox genes are a group of genes that encode transcription factors which play a critical role in the development of structures along the anterior–posterior axis of animals. The 3D arrangement of Hox genes is well documented to influence their spatial and temporal expression during development [60, 73–75]. Modifications in the physical proximity and resulting expression patterns of these genes are associated with morphological and developmental evolution in animals [76–79]. Currently, the genomic location of Hox genes is known for a select few species in 8 out of the 15 major clades that encompass spiralians; however, it is important to note that within these groups, the Hox cluster configuration may vary. Information on the Hox genes in the clades Chaetognatha, Gnathostomulida, Micrognathozoa, Gastrotricha, Entoprocta, Cycliophora and Bryozoa is still lacking. Hox gene content and specific expression pattern in spiralians is reviewed in [80]. Interestingly, unlike the vertebrate Hox clusters, the spiralian Hox clusters show a very different density regime. In vertebrates, Hox clusters are more dense in their coding gene content than other microsyntenic regions. In contrast, in invertebrates, Hox clusters show low gene density regime [68]. This highlights likely very different topological constraints on the Hox clusters in these animal groups.

The diversity in both content and genomic linkage of the spiralian Hox genes is thought to contribute to their divergent body plans [80, 81]. Notably, while most animals have a cluster of Hox genes organised in a linear array on a single chromosome, some spiralians have Hox genes distributed across different chromosomes. For instance, in rotifers (in the group Gnathifera) and free-living planarian flatworms (in the group Platyhelminthes), Hox genes are not present in a genomic cluster which correlates with an absence of temporal and spatial collinear transcription of their Hox genes, influencing their developmental programme [82, 83]. Furthermore, the genome assembly of the dicyemid *D. japonicum* yielded just four Hox genes in this species which are also dispersed throughout the genome [24, 84]. To date, however, no Hox gene expression data are available for any species within the Dicyemia. The nemertean *Notospermus geniculatus* is thought to lack a Hox cluster; however, this could be an artefact of a scaffold-level genome assembly [85] and transcriptomic studies in other nemerteans show that spatial collinearity in expression seems to have been retained in this clade at least during juvenile development [86, 87].

In contrast, research in Annelida and Mollusca show that although Hox genes are present in clusters in most species studied and exhibit spatial and at least sub-cluster temporal collinearity [5, 7, 88–92], this is thought to be the ancestral state in both clades, with the exceptional annelid groups Clitellata and Orthonectida having recently lost their Hox cluster organisation (e.g. *Helobdella robusta* reported in [3]). Even the miniature genome of *D. gyrociliatus* (Figure 1) has a conserved Hox complement, and exhibits spatial collinear expression, although temporal collinearity has been lost in this species. This is intriguing as it appears that genome topological organisation has not been greatly affected by the drastic reduction in genome size, unlike in other species that exhibit genome reduction [6].

The large differences in the spiralian genome sizes may put different emphasis on the role of genome topology in developmental gene regulation. For example, the cephalopod mollusc *E. scolopes* has very large intergenic distances (1 to 2.6 Mbp) in its Hox cluster, which are the largest known inter-Hox gene separators [25]. Intergenic regions between Hox genes are also devoid of any other genes [25]. Regulation over such large distances may also be present in other previously reported and still-enigmatic large cephalopod gene clusters, such as the protocadherin (PCDH) and zinc finger (C2H2) clusters [10, 28]. Recent papers on protocadherin cluster regulation in mouse [93, 94] highlight both the importance of cohesin in achieving long-range interactions in the PCDH alpha gene cluster and the stochastic expression of PCDH genes within. Similar processes can be expected to have a role in cephalopod genomes. While no functional data on the role of cohesin exists for spiralians yet, these insights provide for testable hypotheses using topological and expression studies (including ‘multiome’ approaches) to test these hypotheses.

Together, new high-resolution chromatin interaction data will help resolve crucial developmental gene regulation in spiralians. Long-range interaction data, including inter-chromosomal contacts, may also shed light on whether spiralian chromosomes show evolutionarily conserved chromosomal-scale interactions as has been proposed recently using 3D modelling approaches [69]. The observation of such ‘spatiosyntenic’ (i.e. genomically distant but physically close) conservation highlights the potential importance of chromosomal-scale 3D folding in maintaining gene regulation that goes beyond the ‘local’ (TAD-level) enhancer–promoter interactions. In addition, this highlights the importance of developing further HiC validation approaches in spiralians, such as chromosomal imaging [95], to test the ‘spatiosyntenic’ hypothesis.

## THE ROLE OF GENOME TOPOLOGY IN THE DIVERSIFICATION OF SPIRALIAN GENE REGULATION

Spiralian evolution has resulted in the evolution of a wide range of clade-specific adaptations, as encapsulated by their huge morphological and behavioural diversity. This makes the clade a perfect system in which to study the effect of genome topology on gene regulation and function. The reason/driver behind such fast pace remains to be characterised. Several studies suggest that reshuffling of TADs can ‘rewire’ CREs to act on alternate genes, resulting in transcriptional changes. Although compelling evidence of this exists in some species [96–98], evidence within the spiralian lineage is lacking. Untangling the evolutionary impact of TAD rearrangements in spiralians is not a trivial task given that it requires a detailed understanding of gene function and expression patterns in the non-model organisms. However, there is some indirect evidence that compartment reshuffling is linked to the evolution of novel traits. For example, one study in the bobtail squid *E. scolopes* uncovered hundreds of microsyntenies linked to coleoid cephalopod-specific traits such as their complex nervous system. HiC data revealed that these locally conserved gene neighbourhoods were often located in TADs [27]. Furthermore, 3D chromosome modelling found that cephalopod-specific microsyntenies were more frequently found on the surface of chromosomes than metazoan, ancestral microsyntenies which were more often localised towards the chromosomal core. The authors interpret this as being reflective of the highly dynamic chromatin accessibility and inter-chromosomal regulation of the clade-specific microsyntenies [27], though more studies will be required to reveal how 3D localisation within and along the nuclear lamina can affect gene expression [99]. It should be noted, however, that mutations in TADs may be neutral and not alter gene expression, as one study in *Drosophila* suggests [56]. Furthermore, mutations in regulatory domains are most frequently selected against, with the breakpoints of synteny arrangements being enriched at TAD boundaries in *Drosophila* [51] and vertebrates [100–102].

As well as through TAD rearrangements, genome topology may play a role in adaptation via more subtle changes in chromatin folding [32], e.g. through the weakening of TAD boundaries and divergent loops [103–105]. Moreover, there is evidence to suggest that the distance between enhancers and promoters could account for variation in species-specific gene expression level [105]. Accordingly, it is of particular interest to understand how this would be affected in spiralian species that have undergone an expansion or reduction in genome size. However, as of yet evidence for the role of small-scale chromatin changes in the evolution of spiralian phenotypes is lacking.

Recently, key papers described evolutionary breakpoints (EBR) in closely related vertebrate species [98, 106, 107]. Among several key findings was the association of EBRs to occur predominantly at TAD boundaries. Counterintuitively, TAD boundaries usually represent condensed chromatin regions. The potential mechanism behind such observed EBR enrichment at TAD boundaries has been suggested to be due to chromatin remodelling during late spermatogenesis stages [106]. This would allow regulatory interactions within each TAD to persist as single evolutionary units that are maintained by selective pressure. To this end, changes in EBR localisation and the resulting impact on gene regulation have been suggested to help drive phenotypic evolution [98, 108].

Several closely related spiralian species can provide for excellent opportunities to evaluate the impact of EBRs on phenotypic diversity and for understanding the effect of genome size on EBRs. Recently, several such genomes within bivalves [7, 8, 109, 110], annelids [5, 6], as well as cephalopods [10, 27] became available. The evolutionary distances that these species span are similar to that of vertebrate species investigated in previous studies [98]. Yet, these spiralian species show vastly different genome sizes (Figure 1) and evolutionary histories, ranging from conservation of the genomic organisation [26] to whole genome duplications [30] and large-scale rearrangements [10]. The ancient coleoid rearrangement in cephalopods and its effects on recombination and EBRs in closely related species of octopuses and squids will be particularly important to compare to the findings obtained for vertebrates following such extensive genomic events [107]. Similarly, but to a much lesser degree, several Robertsonian fusions have impacted the genomes of other spiralian species, particularly in species of closely related annelids [26]. As such, these fusions may have had substantial impact on recombination forces affecting modality of gene regulation in these clades. Together, topological data for many of these spiralian clades may help link organismal adaptations these species have experienced to the conservation and turn-over of their topological genomic features.

## CONCLUSION

To conclude, it remains to be seen how significant genome topology data will be in unravelling the different modes of gene regulation in spiralians. In this review, we describe how this role will likely depend on the genome size and gene regulatory modality resulting from the species’ evolutionary history. The plethora of phenotypic traits that emerge in spiralians and the diversity of the underlying developmental programmes has provided for fruitful ground for many evo-devo studies. The addition of more high-quality reference genomes and 3C data to studies such as these can reveal whether genome topology puts increased emphasis on distal enhancers in gene regulation or how neighbouring TADs can contribute to gene regulation at TAD boundaries. As genome-wide methods for genome topology assessment as well as functional tools such as CRISPR-Cas9, and high-resolution expression studies such as multiomics, become readily available, spiralians will likely develop into valuable emerging systems for the study of the role of genome topology in their evolution.

In addition, as more chromosomal-scale genomes including genome topology data become available for this clade, the investigation into the variety of molecular mechanisms can take off. Transposable element-driven genome size differences in closely related species of molluscs, polychaetes, as well as whole genome duplications and rearrangements e.g. in cephalopods have had profound effects on their genomes and topology. So far, clear genome topological structures exist in the few spiralian (primarily, cephalopod) genomes where this has been investigated. While the general genome topology and compartmentalisation (i.e. TAD presence) looks similar to other bilaterian genomes, how those structures are maintained during genome expansion, contraction, across development and whether they change between tissues or cell types will be a crucial emerging topic in the next few years. Taken together, insights into the function of genome topology from spiralians may be crucially informative not only in the understanding of their evolution, but also in the function of topology in well-established model organisms.

**Key Points**

- Spiralians comprise one of the most species-rich, morphologically and genomically diverse clade of animals.
- While ample progress has been made at establishing several spiralian model systems, little is known about how their genomic organisation may impact gene regulation.
- This review discusses recent progress in the field of spiralian genome evolution and topology, and outlines several testable predictions for the effect of genome topology on gene regulation in spiralians.
- In particular, the review proposes that the often very dynamic genome expansion and contraction has played a central role in the emergence, modification and degradation of topologically associating domains (TADs) in spiralian genomes and species-specific adaptations.

## Data Availability

Not applicable.

## Author Biographies

**Thea Rogers** received her BSc in zoology from Cardiff University (2017) and her PhD in evolutionary genomics from the University of Sheffield (2022). Currently, she is working as a postdoctoral scientist in the Simakov Lab at the University of Vienna. Her prevailing research interest lies in understanding the role of gene regulation and selection in the evolution of biodiversity.

**Oleg Simakov** received his PhD at European Molecular Biology Laboratory Heidelberg in 2013. He completed postdocs at the University of Heidelberg and Okinawa Institute of Science and Technology before starting a lab at the University of Vienna in 2017.
